# Properties of Tool Steels Printed by Directed Energy Deposition Process on S45C Base Metal

**DOI:** 10.3390/ma13225068

**Published:** 2020-11-10

**Authors:** Sungjong Choi, Hochan Kim, Jihyun Sung, Dongmok Lee, Jongdock Seo

**Affiliations:** 1 Education and Research Center for Reliability, Andong National University, Andong 36729, Gyeongsangbuk-do, Korea; dgjches@anu.ac.kr; 2 Department of Mechanical and Automotive Engineering, Andong National University, Andong 36729, Gyeongsangbuk-do, Korea; 3 Daegyeong Division, Korea Institute of Industrial Technology, Daegu 42994, Korea; jsung@kitech.re.kr; 4 R&D Center, Maxrotec Co., Ltd., Daegu 42703, Korea; dmlee@maxrotec.com; 5 R&D Center, SHIN YOUNG Co., Ltd., Yeongcheon-si 38899, Gyeongsangbuk-do, Korea; jdseo@shym.co.kr

**Keywords:** metal 3D printing, additive manufacturing, high-strength steel, hot stamping, trimming die, tensile strength

## Abstract

We present a fundamental study on the development of trimming dies at room temperature for the hot-stamping process using directed energy deposition. Specimens of G and F materials were fabricated by machining 3D-printed blocks. The hardness of G-layered specimens was slightly higher than that of F-layered specimens, reaching approximately 700 HV at the surface. The G-layered specimens consisted of columnar and equiaxed dendrites, whereas the F-layered specimens mainly consisted of equiaxed dendrites. Spherical pores were observed inside the layered cross section, whereas relatively large irregular-shaped cavities were observed in layered boundaries. The tensile strengths of the G-layered and F-layered specimens were approximately 1800 and 1650 MPa, respectively. During bonding strength tests on an area bonded with S45C base metal, a fracture occurred in one case because of the lack of fusion at the boundary, and the F-layered specimens showed a lower strength than the G-layered ones. During wear tests on a quenched 1.5 GPa-grade aluminized steel plate, the F-layered specimens showed lower wear loss. However, the G-layered specimens showed better wear resistance during wear tests on a 1.5 GPa-grade electrogalvanized steel plate. These findings serve as fundamental data for additive manufacturing processes using tool steels of high-strength materials with high melting points.

## 1. Introduction

High-strength steel has attracted considerable attention in the automobile industry owing to its high specific strength, and it is extensively used for manufacturing automotive body parts such as center pillars. High-tensile-strength steels can provide the desired strength owing to the martensite particles within the structure of the steels that are developed by quenching and hot forming. Accordingly, hot-stamping processes have been developed for a wide variety of applications [1]. However, it has been reported that the shearing or trimming of a material cooled down to room temperature after hot forming using a die causes damage and life reduction in dies and increases product defects. The primary cause is known to be the quenched structure developed through the hot-stamping process. Therefore, manufacturers generally rely on laser cutting for shearing materials after hot stamping, despite its disadvantages of low productivity and high unit cost [2]. In processes such as additive manufacturing (AM), 3D printing, and freeform fabrication, objects are produced by depositing materials based on 3D model data. Repetitive lamination is widely used in such processes [3].

Currently, industries are focusing on sustainable manufacturing to cope with environmental and climate changes [4]. In particular, additive manufacturing is attracting attention as a process for manufacturing lightweight, high-efficiency parts [5]. In transportation such as automobiles, the use of renewable energy and weight reduction to improve fuel efficiency is being studied using additive manufacturing technology [6]. Technological maturity in using metallic materials is achieved owing to the development of simulation tools for analyzing the stress caused by cooling and contraction [7] and controlling the energy supply in real time [8]. Metallic materials are being studied to develop more diverse combinations of these materials, such as composite or hybrid materials [9] comprising more than two types of metals [10]. In addition, research is underway on tool steels that are not frequently used because of their high melting point or high thermal stress [11]. Various studies are being conducted to determine the optimal process parameters for lamination of tool steels [12], find hybrid manufacturing techniques with multiple additive processes [13], and develop post-treatment techniques involving heat treatment [14]. When molding metals through additive manufacturing, molds for hot stamping with conformal cooling channels [15] are considered highly advantageous [16,17]. In addition, to increase the manufacturing efficiency during molding, hybrid manufacturing methods with cutting processes are considered one of the mainstream methods [18]. Developing shear molds for punching [19] and trimming dies for metallic materials [20] are also being actively carried out.

However, additively laminated tool steels exhibit low strength owing to various defects such as pores and deformation due to residual stress. Various studies have examined the pores and cavities occurring in metallic laminated materials, and some of the interesting research results are as follows.

DebRoy et al. classified the defects formed inside metal laminates into keyhole-induced porosity, gas-induced porosity, and pores formed owing to the lack of fusion. They showed that gas-induced porosity is spherical and is generated by the inflow of the shielding gas into the pores present inside the powder [21]. KURIYA et al. examined the degree of pore generation according to changes in the laser power and input energy density and found that the shielding and carrier gas components could be detected by analyzing the gas components inside the pores [22]. Ng et al. showed that gas porosity increased as the laser power, powder feed rate, and shielding gas inflow increased; further, as the powder feed rate increased, the lack of fusion decreased, but as the lamination rate increased, the powder feed rate decreased [23]. Zhong et al. found that using powder drying treatments, high laser power, fine powders, and powders with uniform particle sizes and shapes can effectively reduce porosity in metal laminates [24]. KURIYA et al. state that increasing the laser power reduces porosity, and when the coagulation time is increased, large pores are easily eliminated owing to the relatively large buoyancy, and small pores are eliminated owing to convective activation, thus resulting in decreased porosity [25]. Dass et al. insisted that there is a global energy density for minimizing the overall porosity, and it is directly proportional to the laser power and inversely proportional to the scanning speed and laser spot size [26]. However, studies on pores, cavities, and defect generation are inadequate, and further studies on the relationships between pore types, contents, shapes and sizes, and strength characteristics are required to optimize the parameters for processing laminated tool steels in industries.

In the present study, we fabricated and analyzed specimens from layered blocks for the development of room-temperature trimming dies used in the hot-stamping process based on the directed energy deposition (DED) method. We examined the feasibility of using the materials in additive fabrication processes for die-based shearing by observing their microstructures and defects, and evaluating their tensile strength, hardness, and wear properties. The test pieces after the hardness tests were examined for identifying defects such as pores with an optical microscope. In addition, scanning electron microscopy (SEM) observations of the tensile fracture surfaces were performed to examine the effects of the types, shapes, and sizes of the pores on the strength of the materials; furthermore, we confirmed that SEM is an effective technique for observing defects in layered metals.

## 2. Materials and Methods

In this study, blocks were fabricated in a representative basic shape using S45C steel, and areas of the blocks that require high hardness were selectively layered with a tool steel. For the fabrication, the DED process [27], which supplies powder materials using a laser as an energy source, was applied as an AM process. The materials used for the DED were powders of GRINDUR (G) and Ferro (F) metals with spherical particle sizes in the range of 50–150 µm. Zigzag (ZZ) and spiral (SP) paths were selected as the scanning paths [28]. Table 1 lists the chemical compositions of the metal powders G and F.

Table 2 lists the laser power, scan speed, layer height, and layer width that were experimentally selected by iterating the fabrication. Multiple specimens were developed from the additive-manufactured parts for the analysis of the physical properties of the deposited layers. We observed the microstructure and defects and performed hardness, tensile strength, and pin-on-disc wear tests.

**Figure 1:**
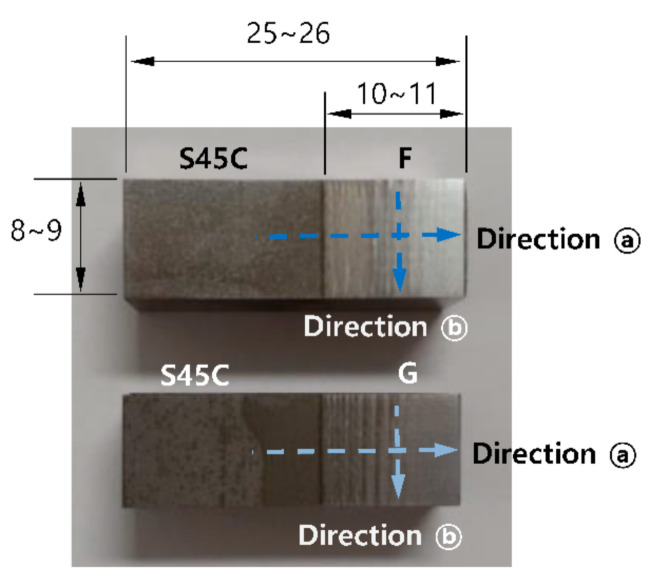
Dimensions and shapes of test specimens used for hardness testing, and microstructural and defect observations (unit in mm).

The samples for hardness measurement were finally finished with #2000 diamond abrasives and washed with acetone just before performing hardness measurements. After measuring the hardness, the specimens were corroded using an etching solution of HNO_3_ (60%, 20 mL), HCl (35%, 10 mL), and CH_3_OH (99%, 20 mL) and observed through an optical microscope to analyze their microstructures and defects. Figure 1 presents a photograph of the specimens, and the building direction coincides with direction ⓐ.

In addition, the tensile fracture surfaces of specimens were observed and analyzed using a scanning electron microscope (manufacturer-Tescan, model-VEGA II LMU, Brno-Kohoutovice, Czech Republic).

**Figure 2:**
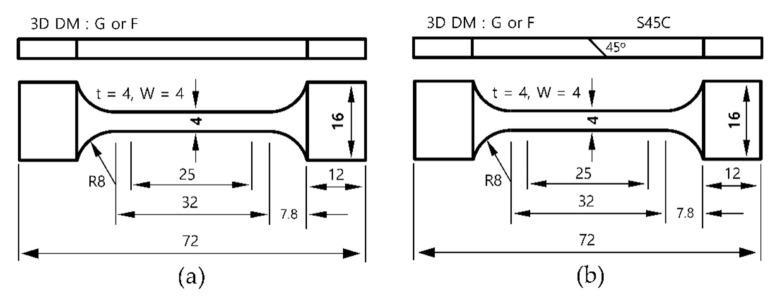
Dimensions and shapes of test specimens used for tensile and bonding strength tests: (**a**) G or F specimens and (**b**) G/F + S45C specimens (unit in mm).

Specimens for the bonding strength tests were fabricated by depositing G or F on the center of S45C to form an angle of 45° at the boundary. Figure 2 shows the shape and dimensions of the specimens fabricated for the tensile and bonding strength tests. The specimens were cut off and fabricated using wire electrical discharge machining, after which they were fixed in a chuck developed for the tests. Elongations were calculated by dividing the grip displacement by the length of the parallel portion because the specimens were too small to be mounted with an elongation gauge. The calculated values were used to develop stress–strain curves.

**Figure 3:**
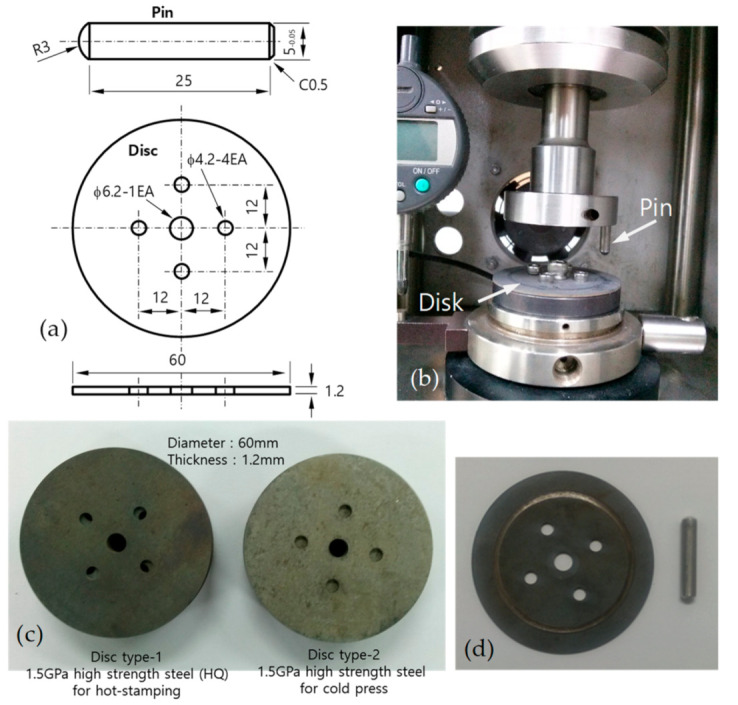
Disc, pin, and testing machine for the pin-on-disc wear test: (**a**) dimensions and shape of the pin and disc (unit in mm), (**b**) testing machine, (**c**) two types of discs, and (**d**) example of a disc and pin after a test (disc: type-1, pin: G).

In the pin-on-disc wear tests, the discs were made with a 1.5 GPa-grade quenched aluminumized steel sheet (22MnB5) with a thickness of 1.2 mm, which is used for hot stamping, and a 1.5 GPa-grade electro-galvanized steel sheet (CR1220Y-EGI, Docol Corporation, Stockholm, Sweden), which is used for cold pressing; the plated thicknesses were 35–50 μm and 12–65 μm, respectively. Figure 3 shows the dimensions and shapes of the pin and disc used for the pin-on-disc wear test, the testing machine (manufacturer-R&B Inc., model-PD-102 Wear Tester, Daejeon, Republic of Korea), two types of discs, and a disc and pin after a test. Specimens were cut off in a disc shape with a diameter of 60 mm from steel sheets using wire electric discharge machining. Pins were machined in the dimensions and shape shown in Figure 3a from the commercially available cold work die steel K340 (Bohler Corporation, Kapfenberg, Austria) and blocks layered with G and F. The contact area R3 was finished using a precision grinder. Wear tests were performed under the same conditions: a vertical load of 29.4 N, rotation radius of R = 22.5 mm, and revolution rate of V = 80 rpm. After the tests, wear loss was obtained by measuring the weight of pins before and after testing with a precision degree of 1/10,000 g.

## 3. Results and Discussion

### 3.1. Microstructure, Defects, and Hardness

**Figure 4:**
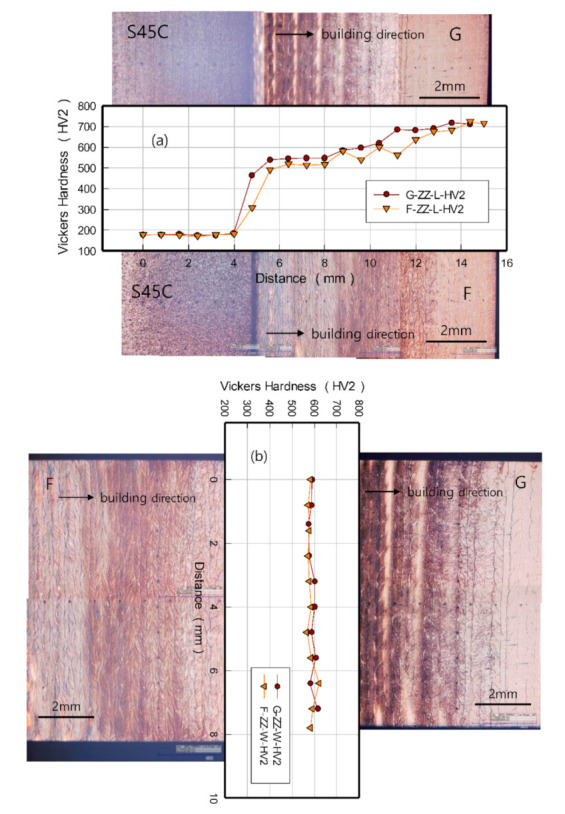
Results of the Vickers hardness test: (**a**) hardness distribution of direction ⓐ in Figure 1; and (**b**) hardness distribution of direction ⓑ in Figure 1.

Figure 4 shows the Vickers hardness and optical microscope images depicting the microstructures of the specimens layered with G and F on the S45C base metal using a 3D printer (manufacturer-Insstek Inc., model-MX-311 Laser-aided Direct Metal Tooling, Daejeon, Republic of Korea). The hardness in direction ⓐ was approximately 180 HV at the boundary with the S45C base metal, but surged to approximately 500 HV in a nearby layered area. The hardness of both materials gradually increased toward the surface of the final layer to approximately 700 HV near the surface. This common tendency is attributed to the differences in the microstructures along the building direction as a result of the differences in the heating cycles.

The hardness in direction ⓑ, measured approximately 8 mm away from the boundary, was 570–620 HV in both specimens; however, compared with the G-layered specimens, the F-layered specimens showed lower hardness by 10–20 HV, but the differences were small.

**Figure 5:**
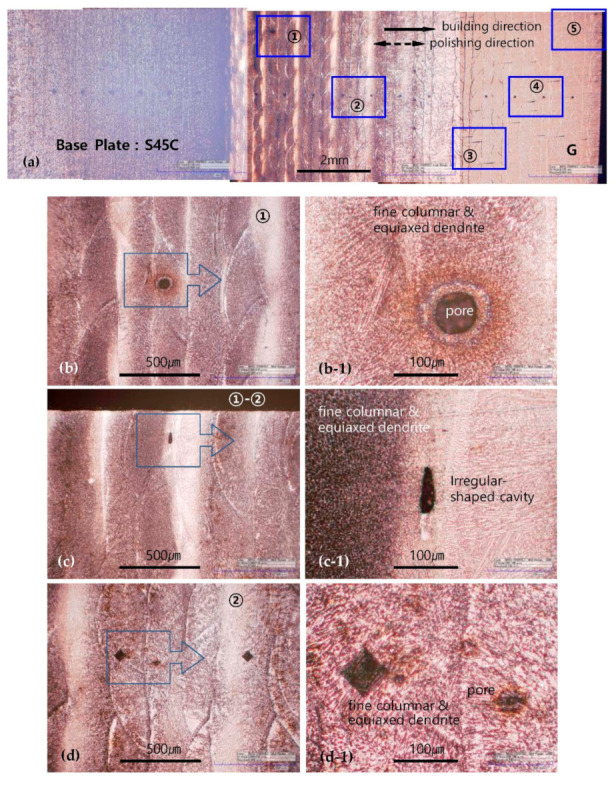

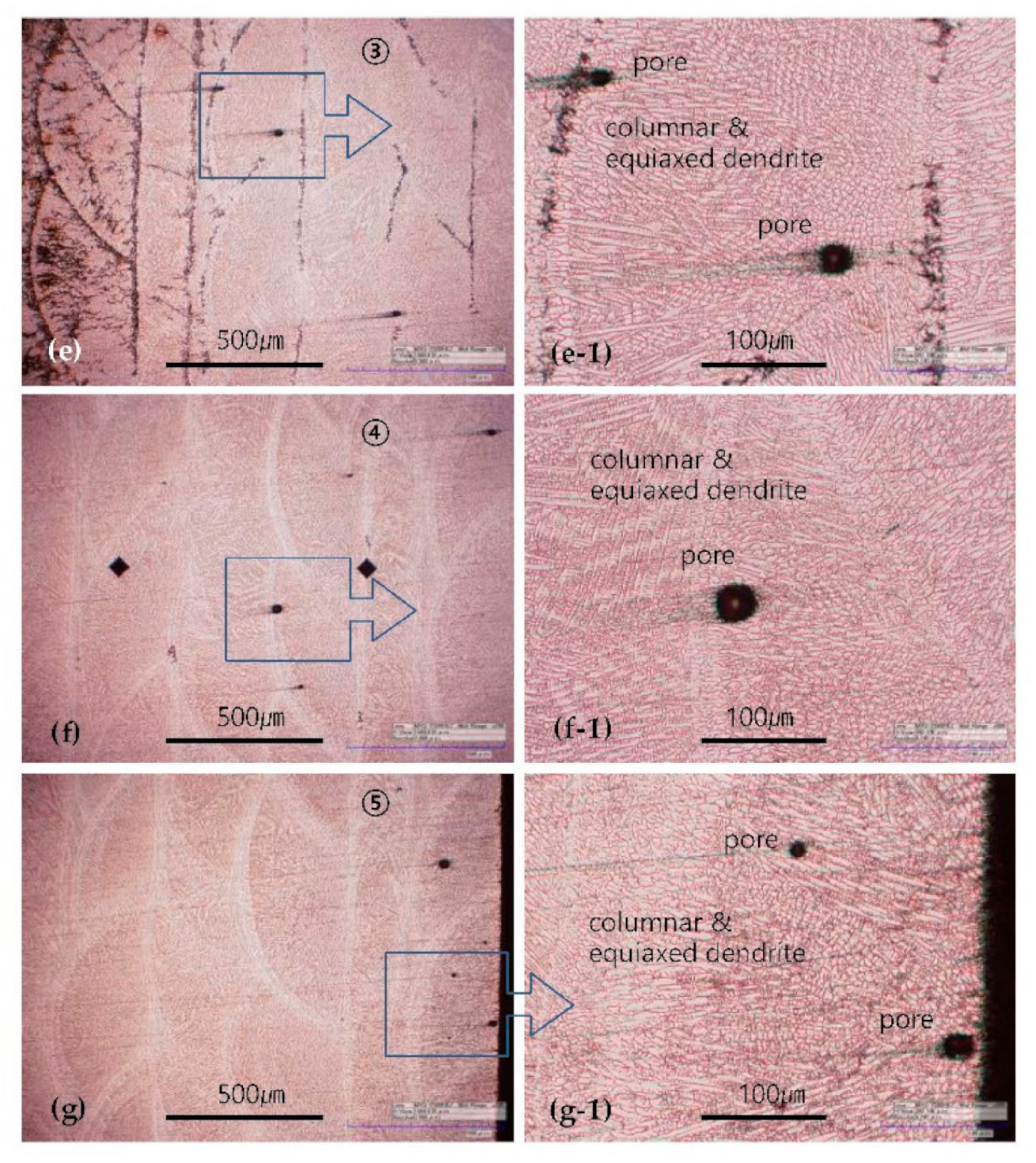
Defects and microstructures of G-layered specimens. Each area in (**a**) is magnified by a factor of 200 in images (**b**–**g**) and by a factor of 800 in images (**b1**–**g1**).

**Figure 6:**
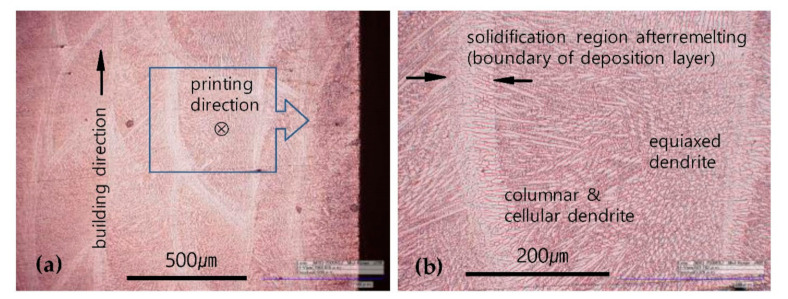
Difference between the layered boundary and internal microstructure in G-layered specimens: (**a**) typical layered structure at low magnification; and (**b**) high magnification view of the rectangle area in (**a**).

Figure 5 and Figure 6 show representative optical microscope (manufacturer-HiROX company LTD., model-KH-7700, Tokyo, Japan) images depicting the microstructures and defects on the surface of the G-layered specimens. Figure 5a shows an image acquired along the entire specimen length. The dark gray area appears stronger toward the initial layer, presumably because of a difference in the heat-treatment effect and microstructure, which is caused by the difference in thermal cycles during continuous layer deposition after the completion of local layer deposition. Each area in Figure 5a is magnified by a factor of 200 in images Figure 5b–g and by a factor of 800 in images Figure 5(b1–g1). The G-layered specimens exhibited numerous tiny spherical pores with a diameter of 50 µm or less in all areas, as shown in the images. In addition, Figure 5a–g clearly show a ZZ scanning path from layered boundaries with striped and colored patterns. Irregular-shaped cavities were also observed, as shown in Figure 5c, but they were rare. The pores mostly had a spherical shape and were present inside the layered cross section, and there were very fine pores with diameters less than 10 µm, appearing as dark brown dots, as shown in (Figure 5(d1)). In the layered structure, equiaxed dendrites were dominantly observed toward the initial layer deposition area with less columnar dendrites, as shown in Figure 5(b1–g1), whereas columnar dendrites were widespread toward the final layer deposition area (surface).

The magnified high-resolution images in Figure 5 were examined in detail to identify the shape of the layered cross sections, boundaries, and inner structures. Consequently, we found that the boundary structures are substantially determined by the process of re-melting, cooling, and re-solidification during continuous layer deposition in the previously deposited upper layers, thereby forming a structure with strong directionality, as observed in a band shape from optical observation [29].

Figure 6 shows representative images of layered cross-sectional structures. The layered boundary formed by the process of re-melting and re-solidifying was larger in scale than the adjacent interior columnar and cellular dendrites, had a different structural directionality, and consisted of equiaxed dendrites mostly toward the center. Given that the specimens were layered in a ZZ path in this study, the columnar or cellular dendrites are predictably present more extensively inside the specimens, as shown in Figure 5(e1–g1).

**Figure 7:**
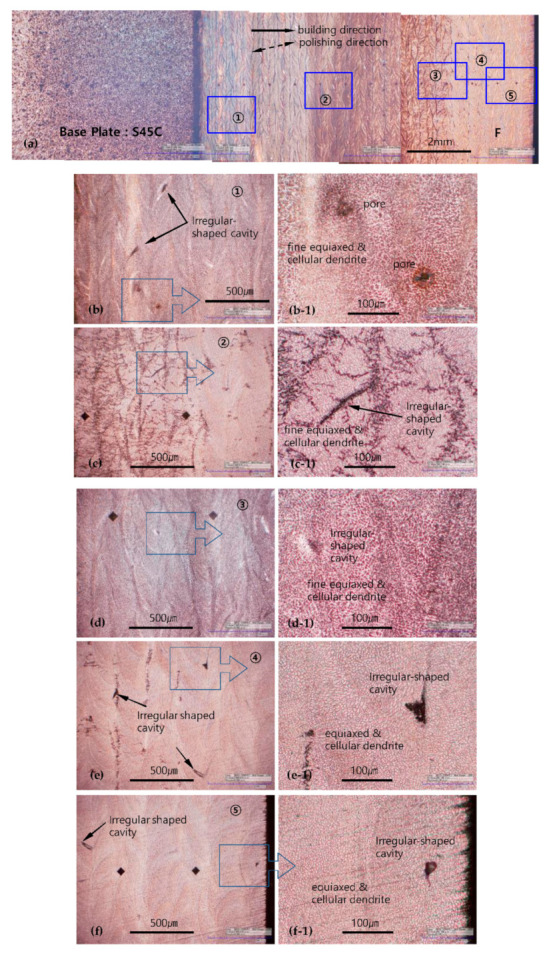
Defects and microstructures of F-layered specimens. Each area in (**a**) is magnified by a factor of 200 in images (**b**–**f**) and by a factor of 800 in images (**b1**–**f1**).

Figure 7 shows the observation results obtained from the F-layered specimens. Images of each area in Figure 7a show the structures along the entire specimen length; the images are enlarged by a factor of 200 in Figure 7b–f and by a factor of 800 in Figure 7(b1–f1).

In the corrosion process, the F-layered specimens displayed a more intense reaction than the G-layered specimens in the same etching solution. Excessive corrosion occurred in a brief span of time, i.e., 2–3 s. Spherical pores and irregular-shaped cavities were also observed in the F-layered specimens, but they were significantly less and smaller than in the G-layered specimens. The microstructure was mainly formed by fine equiaxed dendrites in all areas, as shown in Figure 7(a1–f1), with a rare local appearance of a mixture of cellular or columnar dendrites, as shown in Figure 7(c1). In the areas that were assumed to be layered boundaries, as shown in Figure 7(e1,f1), the size and direction of the boundaries appeared to be slightly different from those of the neighboring dendrites, but the boundaries were not clearly distinguishable as in the G-layered specimens. The layered boundaries appeared quite clearly in the low-resolution images, but were not sufficiently clear to be distinguished in the high-resolution images.

**Figure 8:**
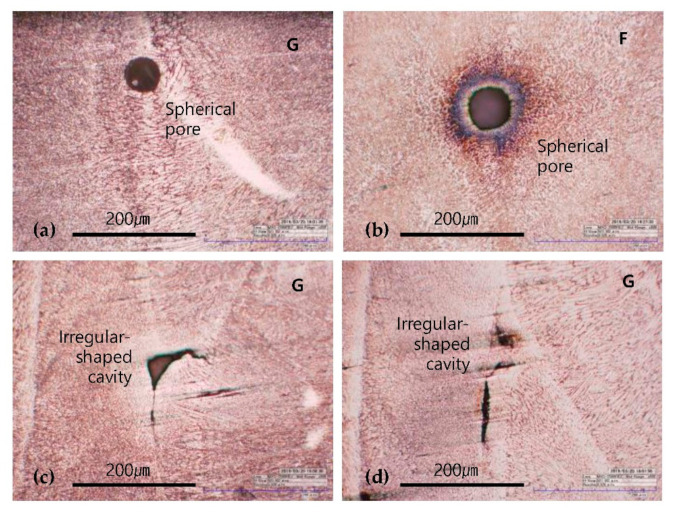
Two typical types of pores on the surfaces of metal deposition layers in G-layered and F-layered specimens. Spherical pores (**a**,**b**) and irregular-shaped cavities (**c**,**d**), for material G (**a**,**c**,**d**) and material F (**b**).

Figure 8 shows the two typical types of pores observed on the surfaces of the G-layered and F-layered specimens. The spherical pores that are in almost complete form, as shown in Figure 8a,b, are known to be created by a mixture of pores contained in metal powders or the keyhole inflow of shielding gas. Several extensive studies have shown that the formation amount of such spherical pores is closely associated with the scale of input energy and scan speed, and that the formation generally occurs in the interior of the deposited layers [22,23,24,25]. Irregular-shaped cavities were mostly found in the layered boundaries, as shown in Figure 8c,d, and they were larger than the spherical pores. Their formation is associated with the hatch spacing and scan speed, and it is known to be caused by the lack of fusion because of a shortage of input energy [26,30].

### 3.2. Tensile Strength of G-Layered and F-Layered Specimens

**Figure 9:**
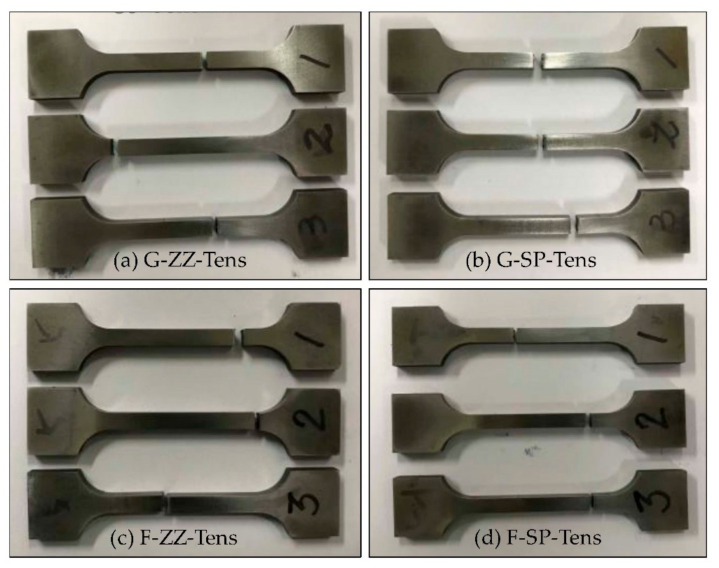
Tensile test specimens fabricated by depositing G and F on S45C base metal (here, ZZ: zigzag, SP: spiral): (**a**) test specimens made from G powder material using a zigzag layer path; (**b**) test specimens made from G powder material using a spiral layer path; (**c**) tests specimens made from a zigzag layer path using an F powder path; and (**d**) tests specimens made from a spiral layer path using an F powdering material.

**Figure 10:**
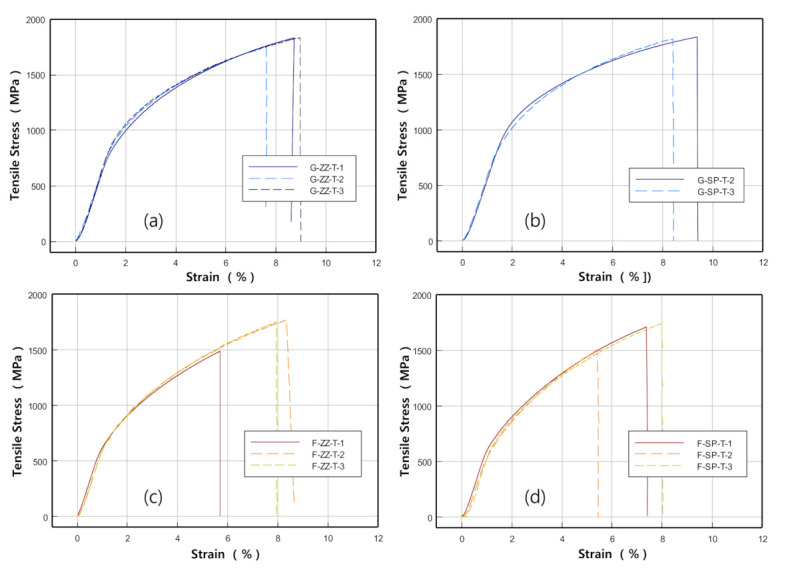
Tensile test results for G and F deposited on S45C base metal. (here, ZZ: zigzag, SP: spiral). The stress-strain curves of (**a**–**d**) are the results of test specimens Figure 9a–d, respectively.

Figure 9 shows photographs of the specimens taken after the tensile tests. Figure 10a,b show the stress–deformation curves obtained from the ZZ- and SP-layered G specimens, while Figure 10c,d are those obtained from the F-layered specimens.

The results indicated that the tensile strength and elongation were higher and significantly larger, respectively, in the G-layered specimens of both the ZZ- and SP-layered types than in the F-layered specimens. When examining the tensile strength and elongation of the same material according to the scanning path difference, the average tensile strength and elongation in the G-layered specimens were found to be slightly higher in the SP-layered types—1828 MPa and 8.9%, respectively—than in the ZZ-layered types—1809 MPa and 8.4%, respectively. However, in the F-layered specimens, the ZZ-layered types showed higher average tensile strength and elongation values—1670 MPa and 7.3%, respectively—than the SP-layered types—1653 MPa and 6.9%, respectively.

**Figure 11:**
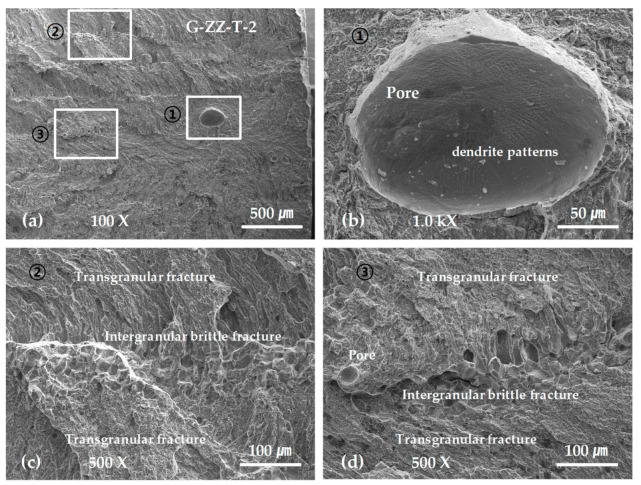
Scanning electron microscopy (SEM) micrographs showing the tensile fracture surface of Table 2. specimen: (**a**) low-scale SEM photograph of near initial fracture; (**b**) magnification view of rectangle ① in (**a**); (**c**) magnification view of rectangle ② in (**a**); and (**d**) magnification view of rectangle ③ in (**a**).

**Figure 12:**
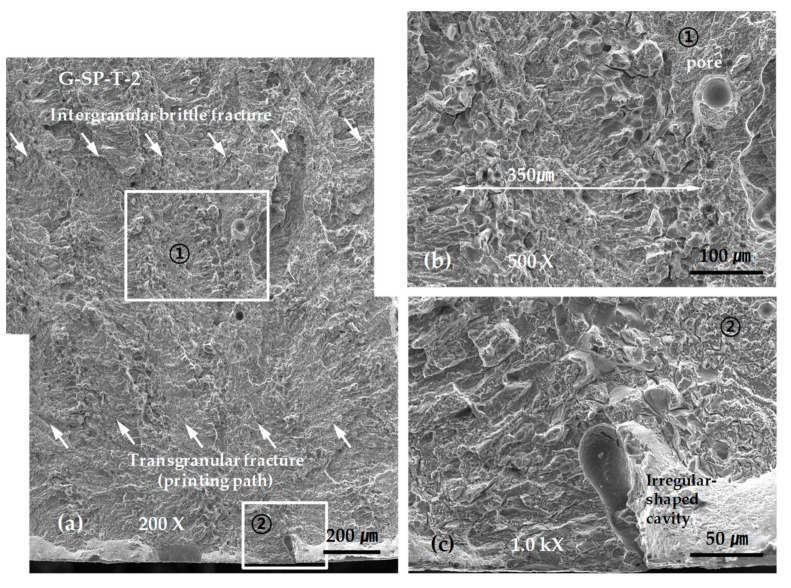
SEM micrographs showing the tensile fracture surface of the G-SP-T-2 specimen: (**a**) low-scale SEM photograph of near initial fracture: (**b**) magnification view of rectangle ① in (**a**); and (**c**) magnification view of rectangle ② in (**a**).

**Figure 13:**
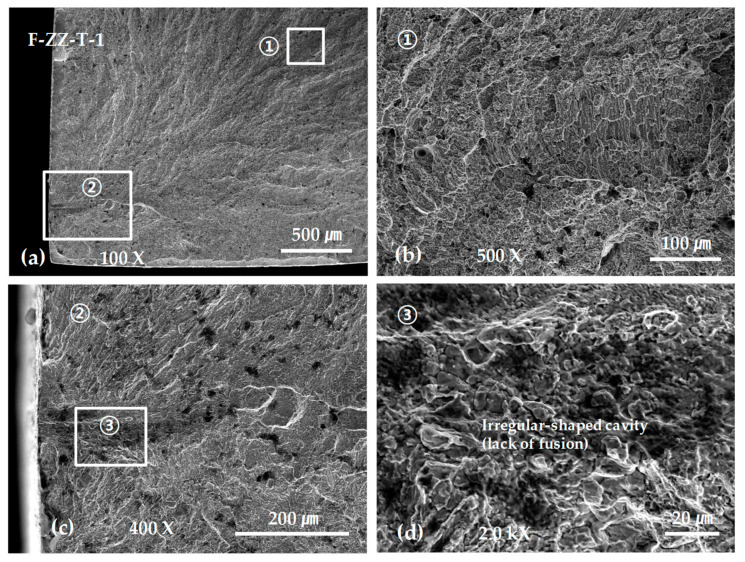
SEM micrographs showing the tensile fracture surface of the F-ZZ-T-1 specimen: (**a**) low-scale SEM photograph of near initial fracture; (**b**) magnification view of rectangle ① in (**a**); (**c**) magnification view of rectangle ② in (**a**); and (**d**) magnification view of rectangle ③ in (**a**).

**Figure 14:**
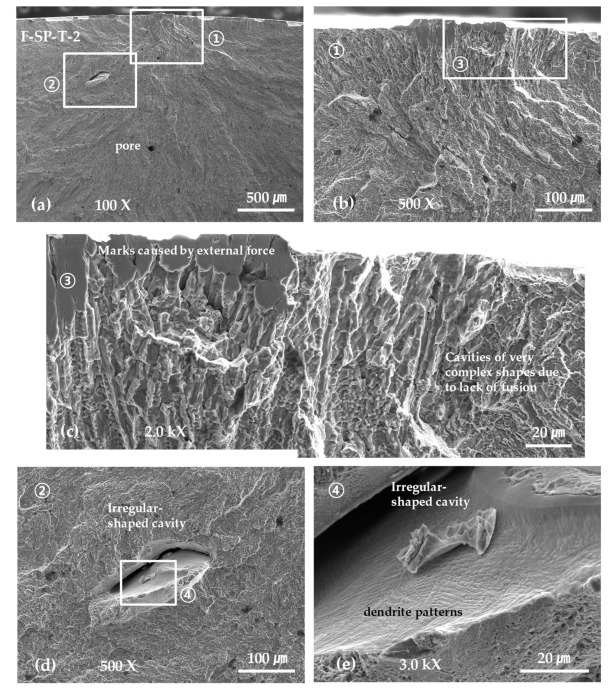
SEM micrographs showing the tensile fracture surface of the F-SP-T-2 specimen: (**a**) low-scale SEM photograph of near initial fracture; (**b**) magnification view of rectangle ① in (**a**); (**c**) magnification view of rectangle ③ in (**b**); (**d**) magnification view of rectangle ② in (**a**); and (**e**) magnification view of rectangle ④ in (**d**).

In addition, the tensile strength and elongation of both the G- and F-layered specimens showed significant deviations, which were particularly severe in the F-layered specimens. Figure 11, Figure 12, Figure 13 and Figure 14 show representative SEM micrographs obtained from the observation of fracture surfaces of the specimens in tensile tests.

Figure 11 shows representative SEM micrographs obtained from the fracture surfaces of the ZZ-layered G tensile test specimens. As shown in Figure 11a,b, the final fractures were grown from a fracture in a large pore with a diameter of approximately 200 µm near the surface. Spherical pores were widely observed in the entire fracture surface area, as shown in Figure 11a,d. Moreover, the specimens exhibited rough fractures, caused by intergranular brittle fractures [31,32], and ductile fractures, caused by transgranular fractures, across the entire fracture surface at regular intervals, as shown in Figure 11c,d.

Figure 12 shows representative SEM micrographs obtained from the SP-layered G tensile test specimens. As shown in Figure 12a, the final fracture was grown from a fracture in an irregular-shaped cavity with a length of approximately 100 µm exposed to the surface. Characteristic areas of intergranular brittle fracture and transgranular fracture were repeated across the entire fracture surface. Moreover, numerous spherical pores were observed in the transgranular fracture area, as shown in Figure 12b,c.

Regardless of the scanning paths, the final fractures of the G-layered tensile test specimens all stemmed from defects near the surface, such as pores. Additionally, the repeated formation of intergranular brittle fractures and transgranular fractures was observed across the entire fracture surface area. It is highly possible that the spacing of the repeated fracture surface morphology is determined by the hatch spacing and thickness of the layered boundaries.

Although the observations are inconclusive, we can presume that the area in which an intergranular brittle fracture occurs contains the layered boundaries formed through re-melting and re-solidification, as explained in the discussion for Figure 6. This is because layered boundaries are likely to be weak owing to a relatively high impurity content and a disconnection or drastic change in the directionality or continuity of the dendrite structures. Another reason is that the spacing of the repeated brittle fracture areas at 300 to 400 µm and the scanning path interval of 500 µm provide effective comparisons, as shown in Figure 12b; moreover, the width of the layered boundary formed by re-fusion and re-solidification is almost identical to the thickness of the layered boundary, which is approximately 60 µm, as shown in Figure 6. In addition, these structural features could not be determined using sectioned specimens for the optical microscope, but they were determined from fracture surface observations using SEM. Hence, SEM image to a fractured surface is better when observing defects in the metal laminates.

It is possible to enhance the tensile strength by reducing the size and content of pores, given that metals in commercial use with a similar composition to the layered materials used in this study have tensile strengths exceeding 2000 MPa and that nearly all the specimens displayed fractures due to defects such as pores around the surface [33,34].

Figure 13 and Figure 14 show representative SEM micrographs obtained from the fracture surfaces of ZZ- and SP-layered F tensile test specimens. Similarly, almost all the F-layered specimens displayed final fractures extending from the initial fracture in a defect near or on the surface. However, the number of defects such as spherical pores was less than that in the G-layered specimens, and no intergranular brittle fractures were observed. This is presumably due to the vagueness of the layered boundaries, as suggested earlier by the structural images, which is thought to result from sufficient energy supply during layer deposition.

Figure 13 shows the SEM micrographs obtained from the fracture surfaces of ZZ-layered tensile test specimens. As shown in Figure 13a,c the initial fracture started from an irregular-shaped cavity with a depth of 200 μm exposed to the surface.

Figure 14 shows the SEM micrographs obtained from the fracture surfaces of the SP-layered tensile test specimens. The images demonstrate that the fracture starts from a considerably large cavity with a complicated shape, a depth of at least 150 μm, and a surface length of 500 μm, and the cavity grows into the final fracture shown in Figure 14a–c. Such pores had a rather different shape from the irregular-shaped cavities that appeared in the G-layered specimens, and were similar to the shrinkage cavities observed in welds and castings. Furthermore, it was difficult to detect the cavity even when observing the polished surface as they were formed along the boundaries of solidified structures with considerable complexity. The reason for such formation is presumably incomplete fusion due to a shortage in energy supply. Figure 14d,e show a large-scale irregular-shaped cavity inside the fracture surface and dendrite patterns formed on the surface of the cavity, respectively.

Examining the SEM photographs of the tensile fracture surface of the F laminated material shown in Figure 13 and Figure 14 revealed the initial failure caused in the irregular-shaped cavities or shrinkage cavities with depths of several hundred µm. This is reflected as a fracture surface shape similar to a fatigue fracture surface, which starts at a specific point on the surface and extends to the entire fracture surface, as shown in Figure 13a and Figure 14a. In addition, it is difficult to observe such sharp and deep defects with an optical microscope targeting a surface polished specimen, but it is clear that observing the fracture surface using SEM is effective for examining the shapes of such defects. Moreover, as shown in Figure 14e, SEM is advantageous for observing the inner surfaces of pores and cavities at high magnifications.

A comprehensive examination of the tensile test results showed that the G laminate had a significantly higher tensile strength and elongation than the F laminate. Considering the constituent elements of the two materials given in Table 2, the sum of the amounts of Cr, Mo, Mn, and Si that contribute to the increase in the hardness is 12.23% for the G material, but 9.6% for the F material. The amount of Mn is relatively large in the G material. We believe that this difference in the amount of Mn causes differences in the hardness and tensile strength owing to different heat-treatment processes and because of repetitive thermal cycles during the lamination process. However, because the F laminate has a lower elongation and tensile strength than the G laminate, it is more affected by relatively sharp and large-scale defects (such as irregular-shaped cavities or shrinkage cavities) on the specimen surface.

Some earlier studies [33,34] evaluating the strength of metal laminates with defects such as pores and cavities report that the strength and elongation gradually decrease as the porosity increases. However, these results were obtained in one kind of identical material. Therefore, as the G and F laminates evaluated in this study are fabricated from different materials, it is not reasonable to simply compare their tensile strengths and elongations. Further research is required in this regard.

**Figure 15:**
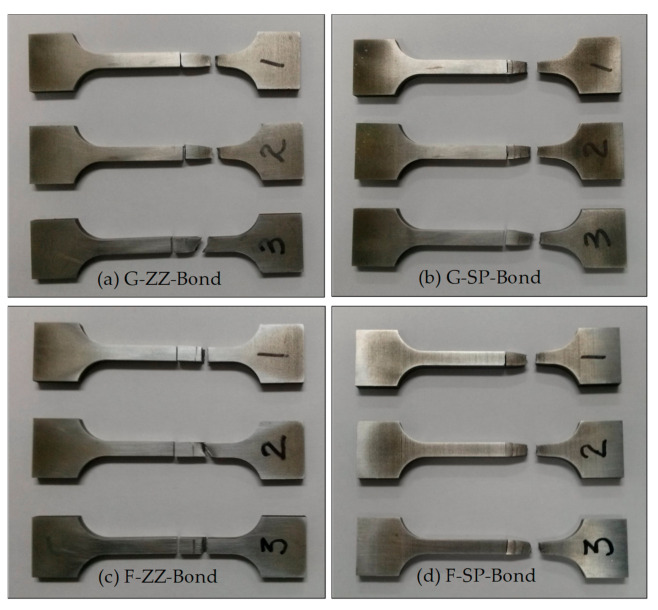
Tensile test specimens with a deposition boundary (S45C + G/F) (here, ZZ: zigzag, SP: spiral): (**a**) test specimens made from G powder material using a zigzag layer path; (**b**) test specimens made from G powder material using a spiral layer path; (**c**) tests specimens made from a zigzag layer path using an F powder path; and (**d**) tests specimens made from a spiral layer path using an F powdering material.

**Figure 16:**
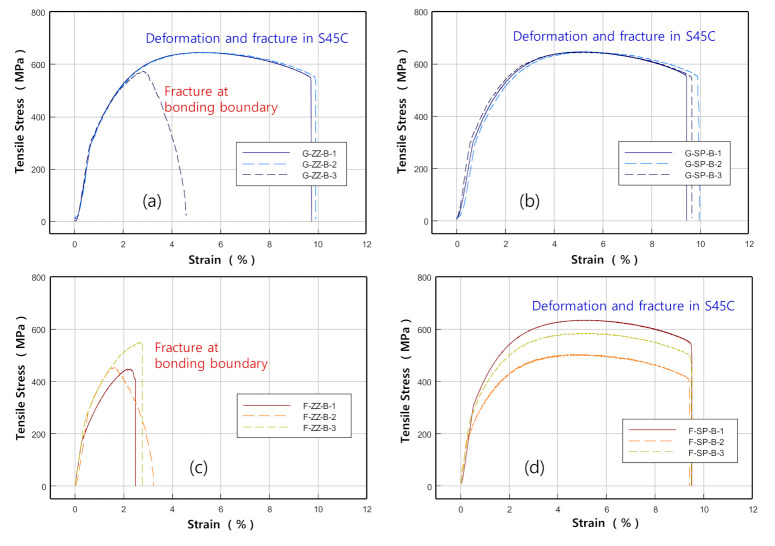
Tensile test results for specimens with a deposition boundary (S45C + G/F) (here, ZZ: zigzag, SP: spiral). The stress-strain curves of (**a**–**d**) are the results of test specimens Figure 15a–d, respectively.

To evaluate the strength of the areas where G and F layers were bonded with the S45C base metal, specimens were fabricated with a 45° inclined bonded area, as shown in Figure 2b for tensile tests. Figure 15 shows the images of specimens taken after the tests, while Figure 16 shows the stress–strain curves obtained from the test results.

The results of testing on the ZZ-layered G specimen G-ZZ-B-3 showed that the initial fracture occurred in a defect on the layered boundary owing to incomplete fusion and grew into the final fracture on the S45C base metal with a maximum stress of approximately 570 MPa, as shown in Figure 16a. The other ZZ-layered and SP-layered specimens sustained their respective final fractures after deformation in the S45C base metal with a maximum stress of approximately 645 MPa.

**Figure 17:**
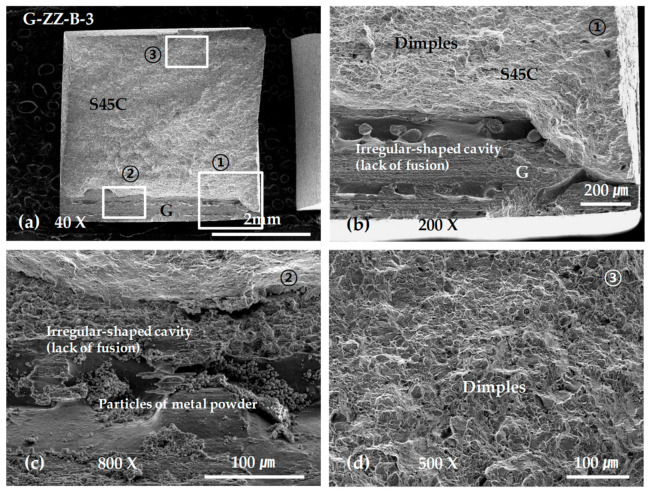
SEM micrographs showing the tensile fracture surface of the G-ZZ-B-3 specimen: (**a**) low-scale SEM photograph of fracture surface; (**b**) magnification view of rectangle ① in (**a**); (**c**) magnification view of rectangle ② in (**a**); and (**d**) magnification view of rectangle ③ in (**a**).

Figure 17 shows SEM micrographs of the fracture surface of specimen G-ZZ-B-3. The image in Figure 17b shows an irregular-shaped cavity in the layered boundary, that in Figure 17c shows unfused metal powder particles, and that in Figure 17d shows typical equiaxed dimples [31] formed in the final fracture area on the S45C base metal.

**Figure 18:**
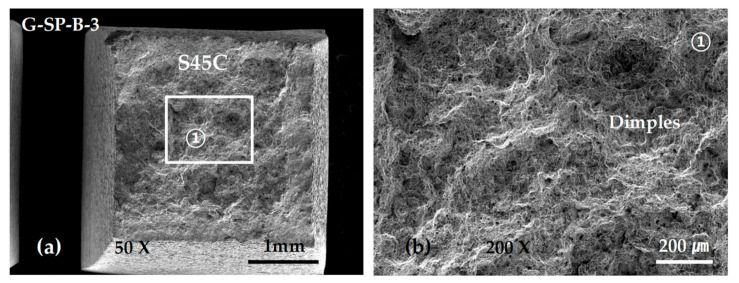
SEM micrographs showing the tensile fracture surface of the G-SP-B-3 specimen: (**a**) low-scale SEM photograph of fracture surface; and (**b**) magnification view of rectangle ① in (**a**).

Figure 18 shows the SEM micrographs of a specimen in which the final fracture occurred after deformation in the S45C base metal. Typical equiaxed dimples were observed in all fractures.

In the bonding strength tests, all the ZZ-layered F specimens showed final fractures preceded by an initial fracture that occurred in a defect due to incomplete fusion on the layered boundaries with the S45C base metal, as shown in Figure 16c.

**Figure 19:**
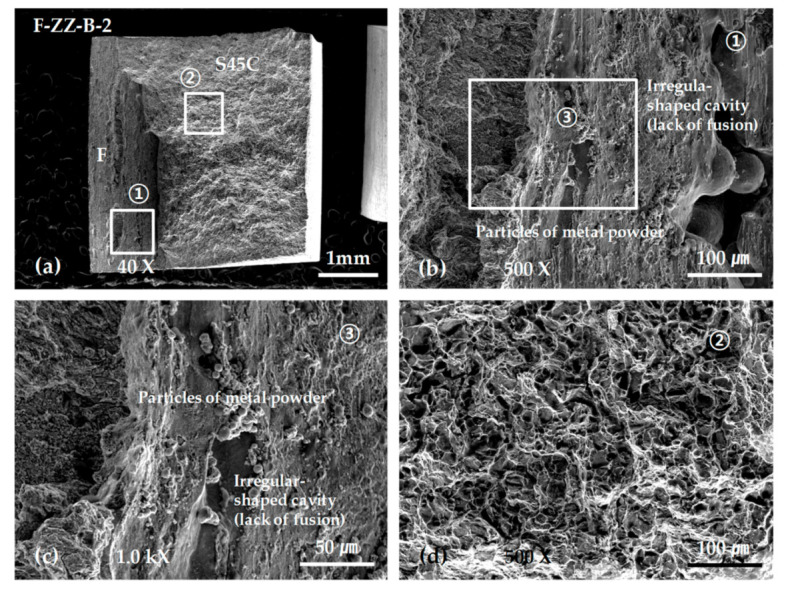
SEM micrographs showing the tensile fracture surface of the F-ZZ-B-2 specimen: (**a**) low-scale SEM photograph of fracture surface; (**b**) magnification view of rectangle ① in (**a**); (**c**) magnification view of rectangle ③ in (**c**); and (**d**) magnification view of rectangle ② in (**a**).

**Figure 20:**
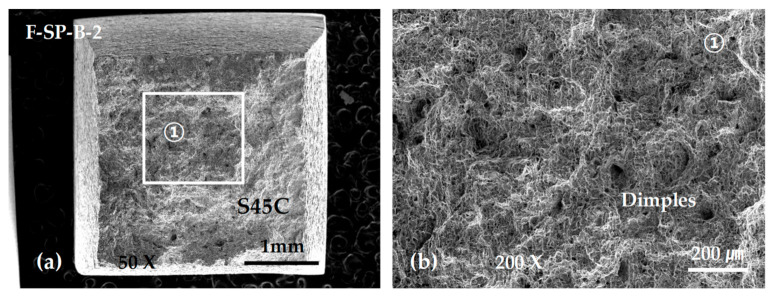
SEM micrographs showing the tensile fracture surface of the F-SP-B-2 specimen: (**a**) low-scale SEM photograph of fracture surface; and (**b**) magnification view of rectangle ① in (**a**).

Figure 19 shows SEM micrographs of the fracture surface of specimen F-ZZ-B-2. The maximum stress was approximately 445 MPa. Magnified views of the image in Figure 19a are shown in Figure 19b,c. The images in Figure 19b,c show irregular-shaped cavities and unfused metal powders generated by incomplete fusion. All the SP-layered specimens exhibited final fractures in the S45C base metal after deformation, as shown in Figure 16d. However, the maximum strength values, which were approximately 635, 580, and 500 MPa, varied significantly, despite the similarity in the stress–deformation curve patterns of the SP-layered specimens. Given that the specimens were developed from three specimens that were extracted from the same layered block, it is possible that the thermal cycles varied significantly among the areas of extraction and that the difference caused a disparity in the microstructural change and mechanical characteristics of the S45C base metal. Nonetheless, such differences did not appear in the bonding strength tests of the G-layered specimens, as shown in Figure 16b. In future studies, it is necessary to conduct a more extensive examination on topics such as the relationship between the physical properties of layered materials and heat-treatment effects accompanied by differences in the scanning path and scanning condition. As shown in Figure 20, all the SP-layered F specimens exhibited final fractures in the S45C base metal. Equiaxed dimples were formed in all fracture surfaces. From the above SEM observation results, we can infer that the sudden decrease in the strength of the G and F laminates joined with the S45C base metal during the tensile tests can be attributed to the formation of pores and irregular-shaped cavities in the joining area and the lack of bonding forces due to the lack of fusion. Preheating of the base metal and supplying sufficient energy could expected to decrease these problems.

**Figure 21:**
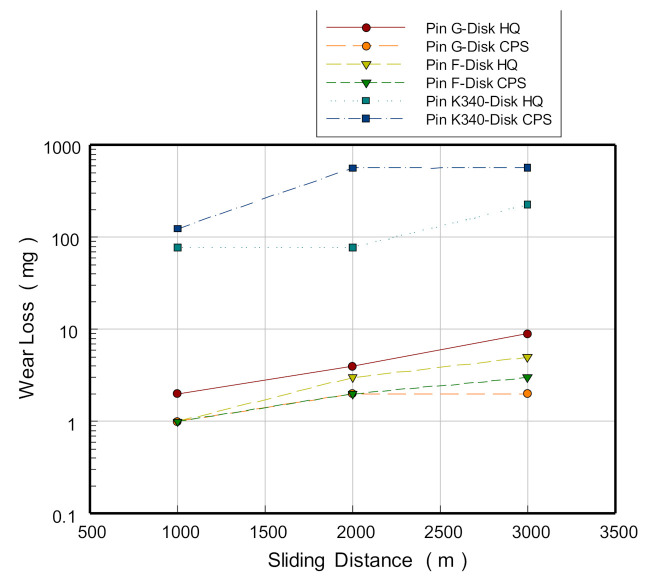
Results of the pin-on-disc wear test. HQ, quenched steel plate used for hot stamping; CPS, steel plate used for cold pressing.

Figure 21 shows the results of the wear tests. In the naming of the discs, HQ refers to the quenched steel plate used for hot stamping, while CPS refers to the steel plate used for cold pressing. The pins developed from K340 sustained more extreme wear in both types of steels, and the degree of wear was at least 100 times more than that in the pins developed from the blocks layered with G and F. In the wear test of the quenched steel plate used for hot stamping, the F-layered pins showed an approximately 40% lower wear loss than the G-layered pins (at a sliding distance of 3000 m). However, in the wear test of the steel sheet used for cold pressing, the G-layered pin showed a wear loss approximately 30% lower than the F-layered pin (at a sliding distance of 3000 m).

In the comparative analysis of wear loss by steel plate type on the same material, a higher wear loss was observed in both the cases where the G-layered and F-layered pins were tested on the quenched aluminum steel plate than in the cases where the pins were tested on the galvanized steel plate. The results demonstrate that the lubrication property of the galvanization layer is more significant than that of the aluminized layer, given the friction and wear occurring in the plating surface of the steel plates in contact with the pin materials in the initial stage of testing.

## 4. Conclusions

We conducted a fundamental study on manufacturing dies through the selective application of metal AM. We performed microstructure and defect observations as well as hardness, tensile strength, bonding strength, and wear tests on specimens fabricated by depositing powder layers of G and F on S45C base metal using the DED method. Based on the findings, it will be possible to manufacture dies in a layered structure using G and F if we accumulate AM technologies and study the optimal conditions for AM that can restrain the formation of large irregular-shaped cavities and reduce the content of defects such as pores. The main findings are summarized as follows.

The hardness of the G-layered and F-layered specimens surged in the layered boundaries of the S45C base metal. It increased near the surface along the building direction and reached approximately 700 HV near the surface. In addition, the G-layered specimens showed slightly higher hardness values than the F-layered specimens. The G-layered specimens mostly consisted of typical columnar dendrites and equiaxed dendrites, while the F-layered specimens primarily consisted of equiaxed dendrites. In addition, both the layered materials had a finer structure near the bonding boundary with the S45C base metal. The defects observed in the layered structures were mostly spherical pores and irregular-shaped cavities. Spherical pores were observed in the interior of the layered cross section, while irregular-shaped cavities tended to be observed at layered boundaries. In addition, the F-layered specimens exhibited less defects than the G-layered specimens. The tensile strength of the G-layered specimens was approximately 1800 MPa, regardless of the scanning path, whereas that of the F-layered specimens was approximately 1650 MPa. However, there were some deviations among the specimens. In addition, repetitive intergranular brittle fractures occurred on the tensile fracture surfaces of the G-layered specimens at a spacing corresponding to the hatch spacing. In the bonding strength tests, there was a case in which a ZZ-layered G specimen exhibited a fracture due to incomplete fusion in the layered boundary with the S45C base metal. However, most specimens exhibited final fractures in the S45C base metal after deformation. All the ZZ-layered F specimens fractured in the layered boundaries with the S45C base metal, whereas all the SP-layered F specimens fractured in the S45C base metal. Nonetheless, there was a large deviation in the maximum (tensile) strength among specimens. In wear tests on a quenched 1.5 GPa-grade aluminized steel plate used for hot stamping, the F-layered specimens exhibited a slightly lower wear loss than the G-layered specimens. In the same tests on a 1.5 GPa-grade electrogalvanized steel plate used for cold pressing, the G-layered specimens exhibited a slightly lower wear loss. The shapes, sizes, and locations of defects such as pores and irregular-shaped cavities formed in the metal laminated material obtained in this study are generally consistent with those reported in many previous studies [21,22,23,24,25,26]. However, repetitive intergranular brittle fractures observed on the tensile fracture surface of the G laminate shown in SEM images have not yet been reported. These intergranular brittle fractures are presumed to occur at the layered boundary, and the formation of these intergranular brittle fractures is presumed to be closely related to the lamination conditions. However, further studies are necessary in this regard. The findings of this study are expected to serve as crucial fundamental data to develop additive metal manufacturing methods using tool steels with high melting points and strengths.

## Figures and Tables

**Table 1:** Chemical compositions of metal powders (weight percentage, %).

Material Type	Cr	Si	C	Mo	Mn	Fe
**G**	9.60	2.40	0.34	-	0.23	87.43
**F**	7.00	0.30	0.35	2.20	0.10	90.05

**Table 2:** Directed energy deposition (DED) conditions for metal 3D printing.

Material Type	Powder Supply (g/min)	Powder Gas (L/min)	Laser Power (W)	Scan Speed (m/min)	Layer Height (mm)	Layer Width (mm)
**G**	5.5	2.5	350	0.85	0.25	0.8
**F**	5.0	2.0	450	0.85	0.25	0.8
